# Outcomes of Hip Arthroscopy for Femoroacetabular Impingement in Chinese Patients Aged 50 Years or Older

**DOI:** 10.1111/os.12688

**Published:** 2020-05-27

**Authors:** Feng Gao, Baiqing Zhang, Bo Hu, Ming Lu, Mingyang An, Yufeng Liu, Yehan Fang, Gang Zhao, Chao Shi, Jingbin Zhou, Yujie Liu, Chunbao Li

**Affiliations:** ^1^ Medical School of Chinese PLA Beijing China; ^2^ Department of Sports Injury and Arthroscopy Surgery National Institute of Sports Medicine Beijing China; ^3^ Department of Orthopedics Chinese PLA General Hospital Beijing China; ^4^ Xinjiang Uyghur Autonomous Region Changji Hui Autonomous Prefecture Qitai County Peoples Hospital Xinjiang China

**Keywords:** Elder patient, Femoroacetabular impingement, Hip arthroscopy, Outcomes

## Abstract

**Objective:**

To investigate the outcomes of hip arthroscopy for femoroacetabular impingement (FAI) in patients over the age of 50 years.

**Method:**

This is a therapeutic case series study. A total of 27 FAI patients over the age of 50 years who met inclusion and exclusion criteria and were being followed up for at least 2 years in the orthopaedics department at our hospital between January 2015 and October 2017 were recruited for a prospective analysis on the outcomes of hip arthroscopy. All patients underwent unilateral surgery. Of the patients included, there were 15 men and 12 women, who were aged 50–74 years old (57 ± 6.4 years). The outcomes were assessed using the visual analog scale (VAS), the modified Harris hip score (mHHS), and the International Hip Outcome Tool (iHOT‐12).

**Results:**

A total of 27 patients were followed up for at least 2 years. The postoperative center‐edge angle, the alpha angle, and the offset decreased significantly compared with preoperative measurements (*P* < 0.01). The mHHS before surgery and at 1 year and 2 years after surgery was 62.19 ± 7.47, 86.70 ± 5.80, and 87.89 ± 5.08, respectively; iHOT‐12 scores were 30.44 ± 4.22, 73.56 ± 3.89, and 73.77 ± 3.72, respectively; VAS scores were 6.07 ± 0.78, 1.93 ± 0.73, and 1.59 ± 0.64, respectively. As compared with the condition before surgery, there was a significant improvement in the mHHS, iHOT‐12, and VAS scores at 1 year and 2 years after surgery (*P* < 0.01). The mHHS score at 2‐year follow up after surgery was higher than that at 1 year after surgery, and the difference observed was statistically significant (*P* = 0.04). One patient with severe acetabular and femoral cartilage damage underwent total hip replacement 11 months after surgery.

**Conclusion:**

Hip arthroscopy considerably improved hip symptoms and function in Chinese FAI patients aged 50 years or older who did not have severe radiographic osteoarthritis. The conversion to THA and complications were low. Strict surgical indications and appropriate surgical strategies lay the foundation for satisfactory postoperative results in elderly patients with FAI.

## Introduction

Femoroacetabular impingement (FAI) is a clinical syndrome in which the anatomic abnormalities of the femoral head and/or the acetabulum result in abnormal contact between the two during hip motion, especially in positions of hip flexion and rotation, leading to cartilage and labral damage and hip pain^1^, ^2^. FAI is more prevalent in Caucasians, and radiographic signs of FAI have been found in over 30% of the population^3^, ^4^, ^5^. Recent studies demonstrated that symptoms of FAI were present in 3% of Caucasians aged 20–49 years^6^. It was reported that the prevalence of radiographic signs associated with a risk of FAI was significantly higher in Caucasians than in Chinese individuals^5^. However, a similar prevalence of radiographic signs of FAI was also found in Chinese individuals^7^. With improved recognition and diagnosis of FAI, increasing numbers of FAI patients are being found in China.

Femoroacetabular impingement is mainly divided into three types: cam type, pincer type, and mixed type. Cam‐type FAI is generally caused by bony proliferation at the femoral head/neck junction. When the hip joint is flexed, the proliferative site comes into abnormal contact with the anterior margin of the acetabulum, leading to injury of the articular cartilage and acetabular labrum. Pincer‐type FAI is generally caused by abnormal development of the acetabulum, which results in excessive coverage of the femoral head/neck, either locally or extensively. Then, when the hip joint moves, the femoral head/neck junction is likely to come into linear contact with the acetabular labrum. Continuous chronic impingement can lead to injury of the acetabular labrum and, hence, lesions of the acetabular cartilage. Characteristics of both cam and pincer‐type FAI are present in mixed‐type FAI. FAI leads to progressive damage within the joint, affecting the acetabular labrum and articular cartilage, and is associated with the development of osteoarthritis of the hip^2^, ^8^.

Surgery has become an established treatment for femoroacetabular impingement syndrome. The aim of such surgery is to reshape the hip joint to prevent impingement. Intra‐articular injuries, such as labral damage, can be resected, repaired, or reconstructed^9^. The choice of open arthrotomy *versus* arthroscopic management remains controversial. As a relatively mature technique, open arthrotomy has shown relatively good results^10^, but it also causes greater trauma and has longer recovery time. Now, with the development of arthroscopy, arthroscopic surgery has become an established treatment for FAI syndrome. Hip arthroscopy can be used effectively to examine and repair the damaged acetabular labrum and cartilage, and can facilitate the management of pincer‐type and cam‐type FAI. Compared with open surgery, hip arthroscopy is safer, has a shorter recovery time, and is associated with fewer complications^11^, ^12^.

Many studies^13^, ^14^, ^15^ show that hip arthroscopy achieved satisfactory outcomes in the treatment of FAI. However, most existing studies have been conducted on younger patients. There are fewer studies on older patients with FAI, even though surgery is also necessary in this cohort when conservative treatment fails. The findings of studies that are available in this respect have thus far been inconsistent. Some have reported that arthroscopy can achieve good clinical outcomes in older FAI patients. Mardones *et al*.^16^ noted that arthroscopic treatment of FAI in patients over 60 years old showed a significant improvement of functional score and pain in most cases. Bryan *et al*., for instance^17^, evaluated the clinical outcomes of patients over the age of 55 years who underwent hip arthroscopy. They found statistically improved outcomes. In contrast, some studies have shown unsatisfactory clinical outcomes following arthroscopy in older patients with FAI. Philippon *et al*.^18^ found that among 153 patients over the age of 50 years, 20% (31 of 153) required total hip arthroplasty (THA) after hip arthroscopy at a mean follow up of 3 years. Domb *et al*.^19^ evaluated 52 patients with a mean age of 54.8 years. They reported that the function scores in the study group were similar to the control group of patients aged 30 years or younger. However, the older patient group had a THA conversion rate of 17.3% at 2 years. Schairer *et al*.^20^ reported that the rate of THA conversion was lowest in the group aged 40 years or younger, at 3.0%. This rate was significantly higher in all older age groups: 16.0% in the 40‐ to 49‐year‐old group, 25.9% in the 50‐ to 59‐year‐old group, 35.0% in the 60‐ to 69‐year‐old group, and 20.7% in the group aged 70 years or older. They noted higher rates of conversion to THA after arthroscopy in older patients. Malviya *et al*.^21^ performed a survival analysis of 6395 patients who underwent hip arthroscopy through the National Health Service in the UK. The results showed that the risk of THA conversion of those aged 55 years or older was five times of that of those aged 55 years or under. Therefore, based on the studies above, whether older FAI patients should undergo arthroscopy is still a matter of controversy.

As the population ages, the proportion of older people in China is increasing. With a rise in the awareness of fitness activities across the country, exercise should become an essential part of their daily activities for more of the elderly in China. FAI is not uncommon in the elderly in China. However, there are quite a few studies that investigate the effectiveness of arthroscopic surgery for older FAI patients. As such, we do not yet know how effective arthroscopic surgery is for older individuals in China.

Therefore, in this study we followed up FAI patients who were aged 50 years or above who underwent hip arthroscopy at our hospital. The purpose of our study was as follows: (i) to investigate the outcomes of hip arthroscopy for FAI in Chinese patients aged 50 years or older; (ii) to discuss the indications for hip arthroscopy in Chinese FAI patients aged 50 years or over; and (iii) to discuss the arthroscopic surgical technique for FAI in Chinese patients aged 50 years or over.

## Materials and Methods

### *Inclusion and Exclusion Criteria*

#### *Inclusion Criteria*

Inclusion criteria were: (i) aged 50 years or over, hip joint pain with or without restricted movement, and clinical findings of joint pathology typically characterized by mechanical symptoms; (ii) unresponsive to nonoperative treatment, including activity modification, oral nonsteroidal anti‐inflammatory medication, supervised physical therapy, and judicious use of intraarticular corticosteroid injections; (iii) positive for hip impingement test upon physical examination (FADIR and/or FABER); (iv) completed radiographic examinations such as anteroposterior X‐ray of the pelvis and frog‐leg lateral X‐ray of the affected hip joint, CT scan of the above positions, and/ or unilateral MRI of the affected hip joint (diagnosis of FAI was based on the following features: acetabular labrum tear, pistol grip deformity, figure‐8 configuration of the acetabulum or the acetabulum excessively covering the hyperplastic bony growth of the acetabular margin, alpha angle ≥50° and offset ≤7.2 mm; (v) positive ultrasound‐guided intra‐articular injection findings; and (vi) hip joint space >2 mm and Tönnis ≤grade 2 in standard weight‐bearing anteroposterior pelvic radiographs.

#### *Exclusion Criteria*

Exclusion criteria were: (i) developmental dysplasia of the hip, with insufficient acetabular coverage upon X‐ray and lateral center‐edge (LCE) angle ≤25°; (ii) severe acetabular retroversion, global pincer upon CT; (iii) a history of hip surgery or hip pathology such as Perthes disease, slipped upper femoral epiphysis, avascular necrosis, or previous hip injury such as acetabular fracture, hip dislocation, femoral neck fracture or rheumatic diseases; (iv) bilateral hip symptoms; (v) body mass index (BMI) greater than 30; (vi) lumbar spine lesions, ankylosing spondylitis or sacroiliac joint diseases; and (vii) contraindications to surgery due to other underlying diseases or cardiopulmonary dysfunction.

### *General Characteristics of Participants*

**Figure 1:**
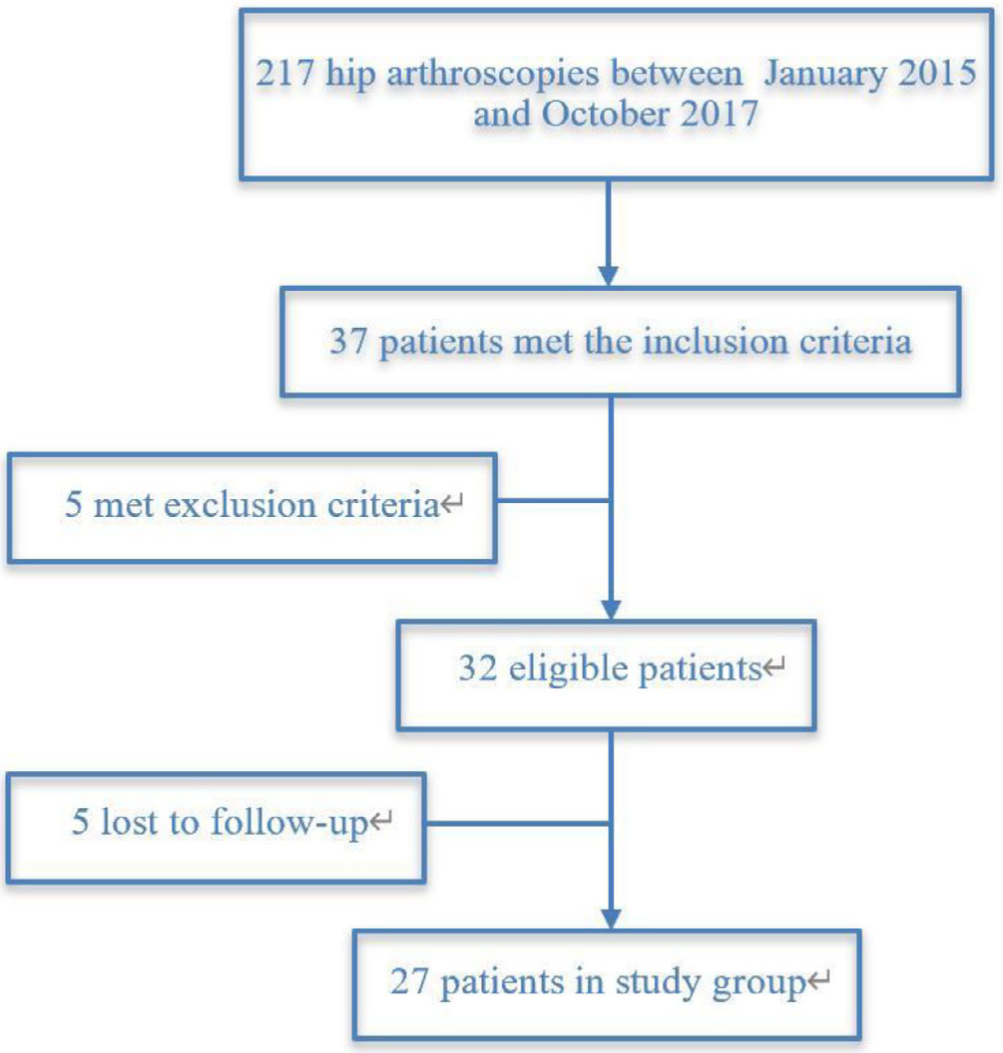
Flowchart showing patient selection for inclusion in the present study.

The protocol was approved by the institutional ethics committee and informed consent was obtained from all patients. Between January 2015 and October 2017, 217 patients underwent arthroscopic hip surgery. We identified 37 patients aged 50 years or older from this group. After application of the exclusion criteria, a total of 32 patients were eligible for review (Fig. 1). A prospective analysis was performed on these patients. Arthroscopy was carried out by one of the senior surgeons (C.L.) in all cases.

### *Surgical Procedures for Hip Arthroscopy*

#### *Anesthesia and Position*

After general anesthesia, the patients were kept in a supine position with both lower limbs placed on the traction bed and immobilized. The perineum was protected. The skin of the affected region was disinfected and a sterile drape was placed over the patient. Traction was applied to the lower limb on the affected side, with adduction and internal rotation of the hip joint.

#### *Arthroscopic Portals*

Under the C‐arm X‐ray, the joint space was retracted apart by 8–10 mm, and a conventional anterolateral approach (AL) was used. The arthroscope was then inserted, and the auxiliary medioanterior approach (MA) applied. The articular capsule was opened and communication between the anterolateral and anterior approaches was achieved.

#### *Processing of the Central Compartment*

The arthroscope was placed into the central compartment of the hip joint. The acetabular labrum, acetabulum and femoral head cartilage, the acetabular fossa, and the round ligament were examined. Debridement or suturing with a suture anchor was performed based on the degree of severity and the quality of the tissue of the damaged acetabular labrum. A distal anterolateral approach (DALA) was established for inserting the suture anchor so that labral repair could be performed, while ensuring surgical safety. If there was excessive acetabular coverage (pincer deformity), the pincer‐type hyperplastic bone on the acetabular side was exposed and resected to restore the normal anatomy. Cartilage damage was graded using the Outerbridge classification system. Radiofrequency ablation was used for trimming damaged cartilage and the degenerative and damaged round ligament, and hyperplastic synovium was resected.

#### *Processing of the Peripheral Compartment*

The retractors on the lower limb were released, and the affected acetabulum was flexed by 35°–45°. The hip arthroscope was placed into the peripheral compartment. The non‐weight‐bearing surface, the femoral head–neck junction, and the hip joint capsule were examined. For severe cam‐type deformity, a T‐capsulotomy incision was made on the articular capsule along the long axis of the femoral neck to fully expose the deformity. The hyperplasia was removed. The impingement was then observed dynamically under the arthroscope. The reshaping was considered successful if the acetabular labrum could pass through the femoral head/neck junction without impingement when the hip joint was 90° in flexion and over 30° in adduction and internal rotation, and abduction and external rotation.

### *Closing the Wound*

In the final step, conventional suture was performed to close the incisions in the articular capsule and skin.

**Figure 2:**
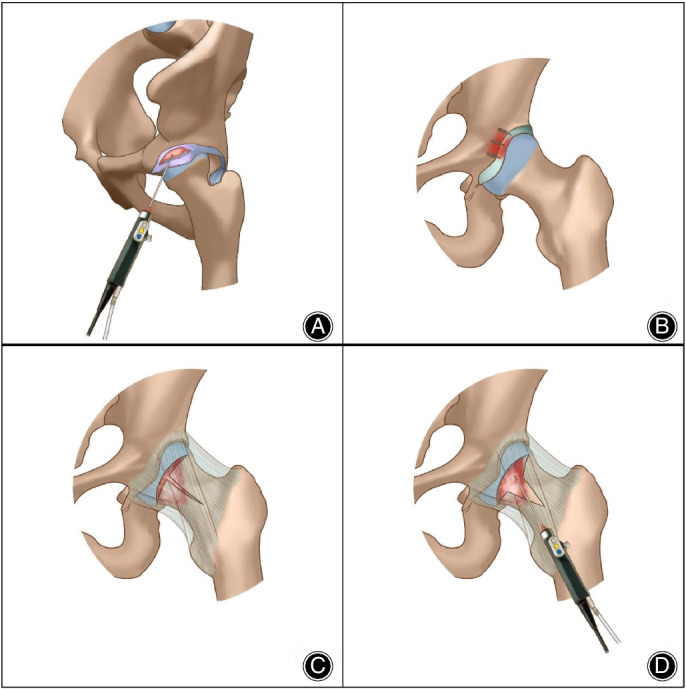
Hip arthroscopic surgical diagrams of the key procedure: (A) probing labrum injury and resecting the pincer deformity; (B) repairing labral with suture anchors; (C) making a T‐capsulotomy incision; and (D) exposing and resecting the cam deformity.

Surgical diagrams of hip arthroscopic surgery are shown in Fig. 2.

### *Postoperative Rehabilitation*

On day 1 after surgery, partial weight‐bearing was prescribed: patients were instructed to stand on two crutches with 30% of their body weight on the operated limb for as long as was tolerable. The hip joint was also passively flexed to 90°. At 1–4 weeks after surgery, patients were allowed to walk with crutches gradually and to fully weight bear within acceptable limits. Active hip flexion of the hip should not exceed 90°, and external rotation and backward extension of the joint should be limited. The gluteus medius, the lumbar back, and the quadriceps muscles were strengthened gradually through strength training. At 5–12 weeks after the operation, full weight‐bearing was possible and a normal range of motion of the joint was restored. Muscle strength and stability around the hip joint will gradually improve through strength training, and daily activities can be resumed. Jogging and stair climbing can also be started.

### *Follow‐Up*

An interviewer assessed all patients undergoing hip arthroscopy preoperatively and postoperatively at 1, 3, 6, 12, and 24 months after surgery. The alpha angle, the offset, the CE angle, and osteoarthritis were examined by X‐ray after surgery for all patients. The hip joint function scores before surgery and at 1 year and 2 years after surgery were also assessed.

### *Observation Indicators*

#### *Labral Treatment Strategy*

Labral tears were repaired when possible. Otherwise, they were selectively debrided until stable. Repair or debridement of labral lesions was recorded.

#### *Outerbridge Classification System*

The system assigns a grade of 0 to IV in the chondral area of interest. Grade 0 signifies normal cartilage. Grade I chondral lesions are characterized by softening and swelling, which often require tactile feedback with a probe or other instrument for assessment. A grade II lesion describes a partial‐thickness defect with fissures that do not exceed 0.5 inches in diameter or reach subchondral bone. Grade III is fissuring of the cartilage with a diameter >0.5 inches with an area reaching subchondral bone. The most severe is grade IV, which includes erosion of the articular cartilage that exposes subchondral bone.

#### *Visual Analogue Scale*

The degree of hip joint pain was evaluated using a visual analogue scale (VAS) score. The degree of hip joint pain was evaluated in all patients using a visual analogue graduated scale. The patient was asked to mark the appropriate position on the graduated scale representing the degree of pain. The score was evaluated according to the patient's mark. The score criteria were as follows: no pain: 0; mild pain, tolerable, not affecting sleep: 1 to 3; moderate pain, mild affecting sleep, still tolerable: 4 to 6; severe pain, unbearable pain, pain resulting in inability to sleep or waking up from sleep: 7 to 10.

#### *Modified Harris Hip Score*

The modified version of the original Harris hip score contains only the patient reported portion. It comprises three domains with eight questions. The domains include pain, function in gait, and function in activities. The total score has a maximum of 91 and is multiplied by 1.1 to give a score out of 100. The modified Harris hip score (mHHS) is scored from 0 (indicating the worst functional outcomes and the maximum amount of pain) to 100 (the best functional outcome and the minimum amount of pain). The interpretation of these scores is as follows: <70 (poor result), 70 to 79 (fair result), 80 to 89 (good result), and ≥90 (excellent result). The mHHS has been widely used in hip arthroscopy.

#### *International Hip Outcome Tool‐12*

The International Hip Outcome Tool‐12 (iHOT‐12) is a 12‐item patient‐reported measure of health‐related quality of life. It is designed to measure the impact of hip disease in active patients and to measure therapeutic outcomes. iHOT‐12 covers four domains (symptoms and functional limitations; sport and recreational activities; job‐related concerns; and social, emotional, and lifestyle concerns). The patient is asked to indicate the severity on a 100‐mm horizontal line (VAS) by marking the line with a slash. Each question has equal weight so that the mean of all questions amounts to the score result, ranging from 0 to 100. A score of 100 indicates full function and no symptoms, whereas a score of zero signifies maximum limitations and extreme symptoms.

### *Statistical Analysis*

Statistical software IBM SPSS 22.0 (International Business Machines, Armonk, New York, USA) was used for statistical analysis. Continuous variables obeying a normal distribution were expressed as *X* ± *S*. Paired *t*‐tests were performed for the comparison of radiographic measurements before and after surgery. Observations at multiple time points before and after surgery were compared by repeated measures ANOVA. *P* < 0.05 indicated a significant difference.

## Results

### *Demographic Data*

The average age of the 27 patients in this study was 57 ± 6.4 years (range, 50–74 years), with 15 male and 12 female patients. There were 5 patients of Tönnis grade 0, 19 patients of Tönnis grade 1, and 3 patients of Tönnis grade 2. Of these patients, there were 5 patients with cam‐type FAI, 2 patients with pincer‐type FAI and 20 patients with mixed‐type FAI, indicating degeneration of the labrum. The mean BMI was 23.2 ± 2.5 kg/m^2^. All patients were operated on unilaterally.

### *Labral Treatment Strategy*

All patients underwent labral repair or labral debridement. There were 9 (33%) patients who underwent labral repair and 18 patients (67%) who underwent labral debridement. Of the 27 patients, 19 had labral tears, which were radial fibrillated (70%). Fibrillated tears had the appearance of a shaving brush and were generally of a hairy appearance at the free margin of the labrum.

### *Outerbridge Classification System*

There were 23 patients (85%) with cartilage damage. According to the Outerbridge classification system, there were 4 patients with grade 0 cartilage damage on the acetabular side (15%), 5 patients with grade I cartilage damage (19%), 12 patients with grade II cartilage damage (44%), 4 patients with grade III cartilage damage (15%), and 2 patients with grade IV cartilage injury (7%); there were 20 patients with grade 0 damage on the femoral side (74%), 3 patients with grade I damage (11%), 3 patients with grade II cartilage damage (11%), 1 patient with grade III cartilage damage (4%), and no patients with grade IV cartilage damage. The Outerbridge classification for all patients is listed in Table 1.

**Table 1:** Outerbridge classification from intraoperative findings

Outerbridge classification system					
	Grade 0	Grade I	Grade II	Grade III	Grade IV
Acetabulum	4 (15%)	5 (19%)	12 (44%)	4 (15%)	2 (7%)
Femur	20 (74%)	3 (11%)	3 (11%)	1 (4%)	0 (0%)

### *Follow‐Up*

**Figure 3:**
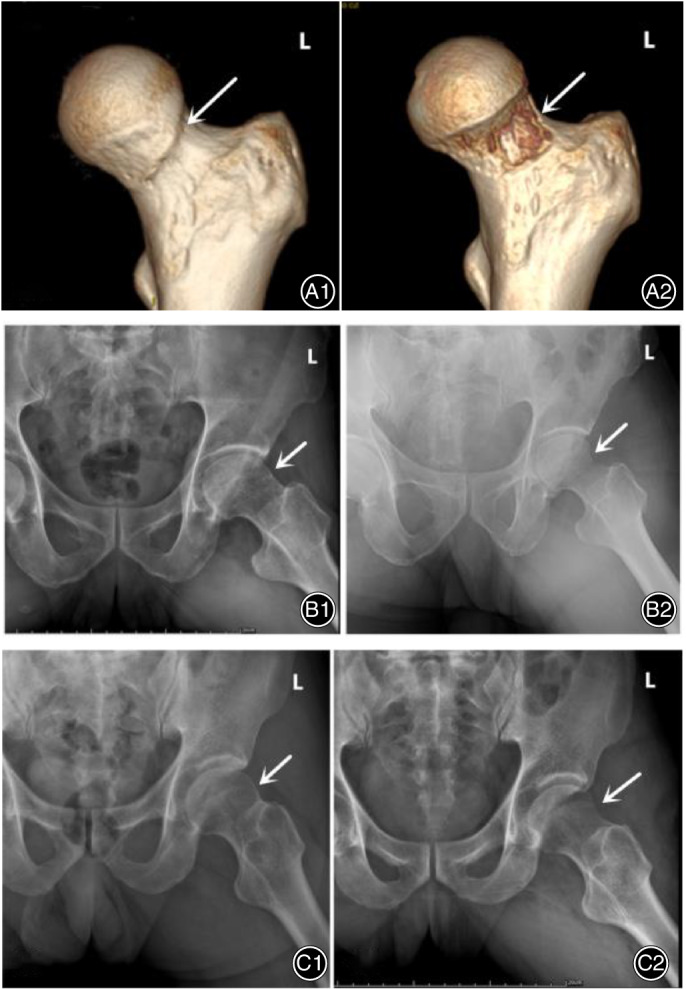
Preoperative CT (A1) and X‐rays (B1, C1) of femoroacetabular impingement (FAI) in 3 patients (A, 54‐year‐old woman; B, 58‐year‐old man; C, 59‐year‐old man); postoperative CT (A2) and X‐rays (B2, C2) of arthroscopy for FAI (arrows indicate changes before and after surgery).

Because 5 patients were unavailable for follow up, the follow‐up rate was 84% (27 patients). The 27 patients were followed up for at least 2 years. One patient underwent conversion to THA 11 months after surgery due to persistent hip joint pain and restricted mobility. The patient who underwent THA was not included in preoperative and postoperative function scoring. The mean CE angle changed from 34.11° preoperatively to 32.59°postoperatively, with a mean decrease in CE angle of 1.52°; the mean alpha angle changed from 58.27°preoperatively to 42.77°postoperatively, with a mean decrease in alpha angle of 15.5°; the mean offset changed from 6.33 preoperatively to 8.46 postoperatively, with a mean improvement in offset of 2.13. The mean CE angle, the alpha angle, and the offset changed significantly as compared to measurements made before surgery (*P* < 0.01). The results showed that the radiographic signs of FAI in the patients was significantly improved and returned to the normal range. See Table 2. Tönnis grading results did not deteriorate in any patients as compared with the preoperative condition. Typical cases are shown in Fig. 3.

**Table 2:** Comparison of preoperative and postoperative radiographic findings

	Before surgery	After surgery
Center‐edge angle	34.11 ± 5.22	32.59 ± 4.13**
α angle	58.27 ± 5.85	42.77 ± 4.97**
Offset	6.33 ± 1.68	8.46 ± 1.17**

Values are expressed as means ± SD; **P < 0.01, *vs* before surgery.

### *Visual Analogue Scale Scores*

The mean VAS score changed from 6.07 ± 0.78 preoperatively to 1.93 ± 0.73 at 1 year postoperatively, with a mean VAS score decrease of 4.41. The mean VAS score changed from 6.07 ± 0.78 preoperatively to 1.59 ± 0.64 at 1 year postoperatively, with a mean VAS score decrease of 4.48. The differences were of statistical significance (*P* < 0.01). There was no significant difference in the mean VAS score between 1 year and 2 years after the operation. See Table 3. The results showed that the pain of the patients was significantly relieved after arthroscopic surgery.

**Table 3:** HSS, VAS, and iHOT‐12 scores before surgery and at 1 year and 2 years after surgery

Scores	Before surgery	1 year after surgery	2 years after surgery
VAS	6.07 ± 0.78	1.93 ± 0.73**	1.59 ± 0.64**
mHHS	62.19 ± 7.47	86.70 ± 5.80**	87.89 ± 5.08**#
iHOT‐12	30.44 ± 4.22	73.56 ± 3.89**	73.77 ± 3.72**

Values are expressed as mean ± SD; **P < 0.01, *vs* before surgery; #*P* < 0.05, *vs* 1 year after surgery. HHS, Harris hip score; iHOT‐12, International Hip Outcome Tool; mHHS, modified Harris hip score; VAS, vector autoregressive scale.

### *Modified Harris Hip Scores*

The mean mHHS changed from 62.19 ± 7.47 preoperatively to 86.70 ± 5.80 at 1 year postoperatively, with a mean mHHS improvement of 24.51. The mean mHHS changed from 62.19 ± 7.47 preoperatively to 87.89 ± 5.08 at 2 years postoperatively, with a mean mHHS improvement of 25.70. The differences were of statistical significance (*P* < 0.01). During the follow up at 2 years after surgery, the mean mHHS improved further still as compared with the condition at 1 year after surgery, and the difference was significant (*P* = 0.04). See Table 3. The results showed that the hip function of the patients was significantly improved after arthroscopic surgery.

### *International Hip Outcome Tool‐12*

The mean iHOT‐12 score changed from 30.44 ± 4.22 preoperatively to 73.56 ± 3.89 at 1 year postoperatively, with a mean improvement of 43.12. The mean iHOT‐12 score changed from 30.44 ± 4.22 preoperatively to 73.77 ± 3.72 at 2 years postoperatively, with a mean improvement of 43.33. The differences were of statistical significance (*P* < 0.01). There was no significant difference in the mean iHOT‐12 score between 1 year and 2 years after the operation. See Table 3. The results showed that the hip function and satisfaction of the patients were significantly improved after arthroscopic surgery.

### *Complications*

During the perioperative and follow‐up period, none of the patients developed complications such as intra‐articular infection, vascular and nerve damage, phlebitis of lower extremities, deep vein thrombosis, or osteonecrosis of the femoral head. There was 1 patient who developed mild scrotal swelling, and 2 patients developed numbness in the root of the thigh on the affected side. However, none of these patients received special treatment, and their symptoms disappeared spontaneously within 3 weeks.

## Discussion

We performed a prospective analysis of clinical and functional improvements after arthroscopy for FAI in patients aged 50 years or older. The result showed that hip arthroscopy could significantly improve the results of VAS, mHHS, and iHOT‐12 scores in Chinese FAI patients aged 50 years or older. The rate of conversion to THA is low.

Hip arthroscopy has become an effective treatment modality for hip pain. Numerous studies have shown that there is improvement in pain and function in patients after arthroscopic treatment for FAI^13^, ^14^, ^15^, ^22^, ^23^. Most of these studies, however, focus on the young active population and the indications for hip arthroscopy in older patients are not well defined. Some clinical studies have shown the opposite results of the present study. Malviya *et al*.,^21^ for instance, performed a survival analysis of 6395 patients who underwent hip arthroscopy through the National Health Service in the UK. The results showed that the risk of THA conversion in those aged 55 years and over was five times that of those aged 55 years and younger. Domb *et al*.^19^ investigated hip arthroscopy in patients with Tönnis grade 0 and 1, and compared the results of patients aged 50 years or older with those aged 30 years and younger. Their study found a higher rate of conversion to THA in patients aged 50 years or older. Schairer *et al*.^20^ reported higher rates of conversion to THA after arthroscopy in patients with osteoarthritis and in older patients. Redmond *et al*.^24^ suggested that arthroscopic treatment of labral tears in patients aged 60 years or older should be considered with caution. Patients in this age group had a high rate of THA conversion within 2 years after surgery. However, only 1 out of the 27 patients recruited in the present study whose treatment was converted to THA after surgery. Significant clinical improvement was achieved in all of the other patients. It is possible that the rate of conversion to THA in our study was lower because we applied stricter selection criteria for surgery in older patients with FAI. Most of the studies mentioned above excluded only elderly patients with a Tönnis grade >2, hip joint space <2 mm, and a history of hip surgery, whereas those with borderline developmental dysplasia of the hips, severe acetabular retroversion, global pincer pathology, or abnormal BMI were not excluded. These conditions may have adversely affected postoperative symptoms, even making them worse in some cases, thus necessitating joint replacement^25^, ^26^, ^27^. Using standard weight‐bearing anteroposterior pelvic radiographs to evaluate hip joint space in our study is more scientific and accurate than using supine anteroposterior pelvic radiographs^28^. In addition to excluding patients based on the strict exclusion criteria for our study, we also performed an ultrasound‐guided intra‐articular injection test on patients and considered arthroscopic surgery only in FAI patients who tested positive. The reason for this is that the pain relief from preoperative intra‐articular hip injections are significantly more likely to achieve positive outcomes after hip arthroscopic surgery^27^.

There are some studies that support the results of this study. Ben *et al*.^29^ investigated the clinical and functional outcomes of arthroscopic labral repair in patients aged above 50 years, and reported marked and significant improvements. Bryan *et al*.^17^ evaluated the clinical outcomes of patients aged 55 years or older who underwent hip arthroscopy. The research has found a 27‐point improvement in mHHS at 1 year after the surgery. Byrd *et al*.^30^ evaluated 21 patients (mean age, 63.2 years) who underwent hip arthroscopy. The average mHHS changed from 57.3 preoperatively to 85.5 points postoperatively and the average iHOT‐12 score changed from 29.8 preoperatively to 67.3 postoperatively. They also noted that patients aged 60 years or older can benefit from arthroscopic labral repair, with only a modest rate of conversion to THA. Our findings are similar to these results, which show an improvement in the mean mHHS from 64.19 preoperatively to 87.89 postoperatively, with a delta of 23.7, for patients aged 50 years or older at 2 years after surgery. The mean iHOT‐12 score changed from 30.44 preoperatively to 73.77 at 2 years postoperatively, with a mean improvement in the iHOT‐12 score of 43.33. The mean VAS score changed from 6.07 preoperatively to 1.59 at 2 years postoperatively, with a mean VAS score improvement of 4.48. A clinically significant (>8 in mHHS)^31^ improvement was observed in all patients who were assessed in this study. During the follow up at 2 years after surgery, the mHHS increased to 87.89 from the previous score of 86.70 at 1 year after surgery, and the difference was statistically significant. The findings above demonstrate that Chinese elderly FAI patients aged 50 years or older can achieve a continuous improvement in prognosis 2 years after surgery. Such a persistent increase in postoperative functional scores has also been reported by Flores *et al*.^32^ They found that hip arthroscopy for FAI yielded a significant improvement in patient outcomes within 2 years of surgery. The improvement mainly occurs within 3 months after surgery, but certain outcomes, such as the ability to return to sports, quality of life, and pain, can continue to improve throughout a 2‐year period.

Grade IV acetabular and grade III femoral articular damage was present in the patient who converted to THA. This was the only case where severe damage in both the acetabular and femoral cartilage was present in our study. This result is similar to that reported by Byrd *et al*.^30^ In their study, all 3 patients whose treatment was converted to THA had combined grade IV acetabular and grade III femoral articular damage. They proposed that when the combination of severe acetabular and femoral cartilage damage was present, the THA conversion rate following hip arthroscopy was the highest, regardless of the age of the patient. Moreover, some other studies suggest that osteoarthritis may serve as a better predictor of worse patient‐reported outcomes and higher THA conversion rates than age^33^, ^34^. Philippon *et al*.^34^ found that joint space ≤2 mm was a criterion for differentiating those who were unfit for hip arthroscopy. This suggests that FAI patients with severe radiographic osteoarthritis are not good candidates for arthroscopic surgery. We agree with these views, and believe that the efficacy of arthroscopic treatment for FAI is related mainly to osteoarthritis, and not age. The patients in our study had no severe radiographic osteoarthritis. Cartilage injury was observed in 23 out of the 27 recruited patients (85%). Although the rate of cartilage damage was high, most injuries were lower than grade II according to the Outerbridge classification system (Table 1). From the results, it is evident that when strict indications for surgery are applied, satisfactory overall postoperative outcomes are possible in elderly Chinese FAI who do not have severe radiographic osteoarthritis, even if a certain degree of cartilage damage is present. Concurrent severe acetabular and femoral cartilage may predict the subsequent conversion to THA.

Furthermore, we believe that surgical technique has an important influence on the effectiveness of the procedure. For instance, we fully evaluated the quality of the labrum and cartilage when operating on elderly FAI patients. In the case of poor labrum quality and/or severe cartilage damage, we carried out selective debridement, as opposed to suturing the labrum. Haddad *et al*.^35^ noted that it seems beneficial to repair an unstable tear in a good quality labrum with the potential to heal to preserve its physiological function. A degenerative labrum, in contrast, may be the source of symptoms and its preservation may result in persistent pain. There is also an increased risk of reattachment failing. Labral debridement requires shorter operative and traction time, and contributes to excellent clinical outcomes. The results of their study do not support routine repair for all labral tears. We agreed with them. Indeed, in our study, degenerative tears in the labrum were present in many cases and labrum suture was suitable in only 33% of patients. In addition, for severe cam‐type deformity, which means cam lesions are too large or too distal to safely visualize and decompress through an interportal capsulotomy, a T‐capsulotomy incision was made on the articular capsule along the long axis of the femoral neck to fully expose the deformity and completely resect it. This reliable and efficient method is used to create and repair the T‐capsulotomy presented by Camp *et al*.^36^ The T‐capsulotomy has the benefits of improving arthroscopic visualization of the femoral neck, facilitating capsular plication, and reducing overall fluoroscopy exposure for the patient and the surgeon. Completely resecting the deformity ensures long‐term efficacy after surgery. In addition, osteoporosis is more common in older patients and care should be taken to avoid the removal of excessive tissue during surgery.

However, this study has several limitations. The main limitation was that there was no control group of patients treated with active rehabilitation or receiving no treatment. The number of cases in this study was also relatively small, and there was a lack of long‐term follow‐up observation. These limitations will be improved upon in further research.

### *Conclusion*

Hip arthroscopy considerably improved hip symptoms and function in Chinese FAI patients aged 50 years or older who did not have severe radiographic osteoarthritis. The conversion to THA and complications were low. Strict surgical indications and appropriate surgical strategies lay the foundation for satisfactory postoperative results in elderly patients with FAI.
